# Congenital Bilateral Absence of the Vas Deferens

**DOI:** 10.3389/fgene.2022.775123

**Published:** 2022-02-11

**Authors:** Zhonglin Cai, Hongjun Li

**Affiliations:** Department of Urology, Peking Union Medical College Hospital, Peking Union Medical College, Chinese Academy of Medical Sciences, Beijing, China

**Keywords:** CBAVD, gene mutation, genetic counseling, reproductive outcome, male infertility

## Abstract

Congenital bilateral absence of the vas deferens (CBAVD) is clinically characterized by the absence of the bilateral vas deferens; the main clinical manifestation is infertility, accounting for 1–2% of male infertility cases. CBAVD may be accompanied by congenital abnormalities in the urogenital system and cystic fibrosis (CF)-related clinical manifestations. CBAVD can develop as a mild manifestation of CF or can be isolated. The main pathogenic mechanism of CBAVD is gene mutation, and CBAVD and CF have a common genetic mutation background. CFTR mutation is the main pathogenic cause of CBAVD and CF, and ADGRG2 mutation is the second most common cause. Although lack of the vas deferens in CBAVD patients causes infertility due to the inability to release sperm, the testes of CBAVD patients have spermatogenic function. Therefore, CBAVD patients can achieve fertility through sperm retrieval surgery and assisted reproductive technology (ART). However, gene mutations in CBAVD patients can have an impact on the ART outcome, and there is a risk of passing on gene mutations to offspring. For CBAVD patients and their spouses, performing genetic counseling (which currently refers mainly to CFTR mutation screening) helps to reduce the risks of genetic mutations being passed on to offspring and of offspring having CF with concomitant CBAVD.

## Introduction

Congenital bilateral absence of the vas deferens (CBAVD) is an important cause of male infertility, accounting for 1–2% of such cases (Hussein et al., 2011). Its main manifestation is the absence of the bilateral vas deferens, wherein the sperm produced by the testes fail to be exported after passing through the epididymis, resulting in male infertility (Bieth et al., 2021). Therefore, CBAVD is often diagnosed due to infertility derived from a lack of sperm and has a certain genetic risk. CBAVD can be one of the symptoms of cystic fibrosis (CF), a human autosomal recessive disease called CF-CBAVD that occurs in more than 95% of CF cases (Chillón et al., 1995). However, CBAVD may not be associated with CF, and in these cases, the disease is referred to as isolated CBAVD (Bieth et al., 2021). In addition to CF-related symptoms, other congenital genitourinary abnormalities, mainly including dysplasia or the absence of the kidneys and seminal vesicles, can accompany CBAVD (Casals et al., 2000; Akinsal et al., 2018). Patients with CBAVD can conceive offspring through sperm extraction surgery and assisted reproductive technology (ART) (Viville et al., 2000; Kamal et al., 2010; Llabador et al., 2015). The pathogenesis for CBAVD is widely accepted to be due to genetic mutations that have a certain heredity in the offspring of CBAVD patients; therefore, CBAVD patients and their spouses need to undergo genetic counseling when considering the conception of offspring to ensure that their offspring have no risk of CF and CBAVD (de Souza et al., 2018). This review provides a detailed introduction to multiple aspects of CBAVD, including its etiology, clinical manifestations, imaging examination, diagnosis, treatment and ART outcomes, and discusses genetic counseling.

## Etiology

There are two views on the pathological mechanism of CBAVD: vas deferens agenesis and vas deferens atresia (Bieth et al., 2021). Although the detailed pathological mechanisms need to be further confirmed, these pathological mechanisms are generally recognized to be caused by gene mutations.

### Cystic Fibrosis Transmembrane Conductance Regulator Mutation

Cystic fibrosis transmembrane conductance regulator (CFTR) gene mutation is the first to be found to be related to CBAVD, and this mutation is considered to be the main causal entity in the occurrence of CBAVD (Kerem et al., 1989; Rommens et al., 1989). *CFTR* is located on the long arm of chromosome 7, the detailed position is 7q31.2, and the total length of *CFTR* is 250 kb (Riordan et al., 1989). It has a total of 27 exons and encodes a protein product comprising 1,480 amino acids (Riordan et al., 1989). The CFTR protein consists of five domains: two nucleotide-binding domains (NBDs), namely NBD-1 and NBD-2; two membrane-spanning domains (MSDs), namely, MSD-1 and MSD-2; and one regulatory domain R (Welsh and Smith, 1993). CFTR is a glycosylated transmembrane protein that acts as a cAMP-regulated chloride ion channel on the apical membrane of many epithelial cells and functions together with several ion transporters, including chloride/bicarbonate exchangers, sodium channels, water channels (aquaporins) and proton exchangers (Na+/H+) (Choi et al., 2001).

With the accumulation of CBAVD and CFTR mutation research in different countries, more than 2000 *CFTR* mutants have been found to be closely related to the formation of CBAVD (Bieth et al., 2021). Among them, some variants, such as ∆F508, IVS8-5T, the (TG)m variant, and M470V, are frequent mutants that contribute to CBAVD occurrence (Chillón et al., 1995; de Souza et al., 2018). Additionally, *CFTR* mutation in CBAVD has the following two characteristics. 1. The frequency of *CFTR* mutants in CBAVD is significantly higher than that in non-CBAVD male infertility. *CFTR* mutants also occur in non-CBAVD obstructive azoospermia and spermatogenetic failure, where the frequency of *CFTR* mutants, such as the 5T allele, is lower than that in CBAVD (Stuppia et al., 2005; Asadi et al., 2019; Rudnik-Schöneborn et al., 2021). Some mutants in CBAVD are not even present in non-CBAVD male infertility. For example, there was no ΔI507 or ΔF508 in nonobstructive azoospermia in a study from India (Heidari et al., 2017). In non-CBAVD male infertile patients in Tunisia, ΔF508 and the 5T allele could not be detected (Ghorbel et al., 2012). 2. The types, frequencies and roles of *CFTR* mutants are significantly different between different races and regions. In terms of mutant frequency, the number of Caucasians with two *CFTR* mutants is significantly higher than that of non-Caucasians with these mutants. In terms of mutant types, the frequency of F508del in Caucasians is significantly higher than that in non-Caucasians, while the frequency of 5T variation is the opposite (Yu et al., 2012). Additionally, it has been shown that mutants in the promoter region of *CFTR* in Chinese individuals are significantly different from those in Caucasians (Bai et al., 2018; Feng et al., 2019). In terms of mutant roles, meta-analysis showed that 5T variation (OR = 8.35, 95% CI = 6.03–10.81) is a risk factor for CBAVD populations in Sydney, France, India, China, Egypt and Iraq, while ΔF508 (OR = 22.2, 95% CI = 7.49–65.79) is a risk factor for CBAVD populations from Slovenia, Iran, Canada, and Egypt. Interestingly, among CBAVD populations from France, Italy, China and Iran, M470V is a protective factor (OR = 0.74, 95% CI = 0.60–0.91) (Xu et al., 2014).

Under physiological conditions and except in sperm, CFTR is expressed in the germ cell cytoplasm and cytomembrane (Teixeira et al., 2013). CFTR expression on the cytomembranes of Sertoli cells and diploid sperm cells of the testes of CBAVD patients is reduced, and CFTR expression in the cytoplasm is lost (Teixeira et al., 2013). Experimental research has also confirmed that the sperm fertilizing capacity is attenuated due to a lack of CFTR-mediated transportation of carbonate ions during the sperm capacitation process (Xu et al., 2007). It has also been shown that ∆F508 and IVS8-5T increased the risk of infertility by 1.63- and 1.69-fold in nonobstructive azoospermia, respectively (Yang et al., 2020). Therefore, in addition to the absence of the vas deferens in CBAVD patients, *CFTR* mutations may be involved in spermatogenesis.

### Adhesion G Protein-Coupled Receptor G2 Gene Mutation

The adhesion G protein-coupled receptor G2 gene (ADGRG2) is considered to be the second mutated gene that causes CBAVD (Patat et al., 2016). It is located at Xp22.13, has 29 exons and produces 10 transcripts, of which the longest transcript is 3.1 kb, which encodes adhesion G protein-coupled receptor G2 (Bieth et al., 2021). ADGRG2 is expressed mainly on the apical lumen membrane of nonciliated epithelial cells of the human efferent duct (Bieth et al., 2021). Studies have found that among CBAVD patients without *CFTR* mutations, some carry ADGRG2 mutations, such as p. C570Y, p. K990E, p. Glu516Ter, p. Cys949AlafsTer81, p. Leu668ArgfsTer21 and c. G118T: p. Glu40* (Patat et al., 2016; Yang et al., 2017; Wu et al., 2020). These mutants are considered to be related to the occurrence of CBAVD. One experimental study confirmed that in the cases with ADGRG2 mutations, the proximal epididymis tissue lacks ADGRG2 protein expression (Wu et al., 2020). This further suggests that the loss of ADGRG2 protein or function caused by ADGRG2 mutation is closely related to the occurrence of CBAVD. In addition, a study found that among CBAVD patients without CFTR mutations, those without renal abnormalities had ADGRG2 mutations; however, ADGRG2 mutations were not detected in those with renal abnormalities (Patat et al., 2016). This suggests that patients with renal abnormalities plus CBAVD may have the same genetic mutation background as that of patients with CBAVD alone but that the genetic background of renal abnormalities is different from that of CBAVD alone.

### Other Genetic Mutations

In addition to the two genes and their mutations mentioned above, several other mutated genes have been screened out and linked to CBAVD. In the Taiwanese CBAVD population, most did not carry CFTR mutations but did have *SLC9A3* mutations and single-copy deletions (Wang et al., 2017). SLC9A3 single-copy deletions cause a decrease in CFTR protein expression, which in turn leads to changes in the structure of the epididymis and vas deferens (Wang et al., 2017). Basic research has also confirmed that knockout of *SLC9A3* in mice can cause atrophy of the vas deferens and disclosure of the seminal vesicle mucosa (Wu et al., 2019). Clinically, *SLC9A3* deletion can increase CBAVD incidence from 3.1 to 37.9% (Wu et al., 2019). Additionally, PANK2 mutations including copy number variations and homozygous deletion were also found in Taiwan CBAVD patients (Lee et al., 2009). Finally, one study suggested that the genetic etiology of CBAVD may be changes in transcriptional and posttranscriptional activity and found that RNA *SCNN1B* and *CA12* mutations were related to CBAVD (Shen et al., 2019).

## Clinical Features

In most cases, CBAVD is a clinical manifestation of CF rather than an isolated event (Chillón et al., 1995; Lewis-Jones et al., 2000); therefore, the clinical manifestations can be diverse (Bombieri et al., 2011). In addition to infertility due to the absence of the bilateral vas deferens, CBAVD may be accompanied by abnormalities of the genitourinary system, mainly including abnormalities in or the absence of the seminal vesicles and kidneys (Casals et al., 2000; Radpour et al., 2008; Cai et al., 2019). The volume of the testis is typically normal or small (Boucher et al., 1999). Semen parameter analysis shows azoospermia, a lower ejaculation volume (<1.0 ml) and pH < 7.0 (Boucher et al., 1999). FSH levels are below the normal range, and the level of each seminal plasma biochemical marker is lower than normal (Boucher et al., 1999). In addition to the abovementioned genitourinary system-related manifestations, CF-CBAVD patients may have remarkable clinical manifestations of CF including recurrent idiopathic chronic pancreatitis; respiratory diseases, such as chronic obstructive pulmonary disease and bronchitis, high-chloride sweat, and sinusitis, among others (Bombieri et al., 2011; Schulz and Tümmler, 2016; Averill et al., 2017).

## Imaging Examination

Strong evidence of the absence of the vas deferens in CBAVD patients relies on imaging examination. Although the aim of the physical examination of scrotum can by met (AbdElnaser et al., 2021), for those with residual vas deferens or fibrous cord-like structures remaining after vas deferens atresia, the diagnosis of CBAVD is often missed. Imaging examination can address this limitation. B-ultrasound is the simplest and most harmless imaging examination method. It can not only clearly show the vas deferens through the transabdominal, scrotal, and transrectal methods (Schlegel et al., 1996; Daudin et al., 2000; Liu et al., 2017) but also detect whether the seminal vesicles, kidneys and other structures are abnormal (Lotti and Maggi, 2015). This method is always used by andrologists clinically during the diagnosis of CBAVD. However, there are certain deficiencies when displaying structures such as the vas deferens and seminal vesicles (Chiang et al., 2013). MRI can also clearly show defects of the internal seminal tract and internal organs of the genitourinary system and provide valuable information on the vas deferens and seminal vesicles that cannot be obtained by B-ultrasound, including the remaining segments of the vas deferens and the remaining seminal vesicles (Chiang et al., 2013). Therefore, in addition to physical examination for the vas deferens, B-ultrasound should also be performed. If the diagnostic results obtained are inconsistent with other clinical manifestations of CBAVD, especially the semen analysis, further MRI examinations should be performed to confirm the diagnosis of CBAVD.

## Diagnosis and Differential Diagnosis

The diagnosis of CBAVD relies mainly on identification of the absence of the bilateral vas deferens through physical examination and imaging methods, and then based on the following semen-related examination results, the diagnosis is further confirmed: low ejaculate volume (<1.0 ml or 1.5 ml are also reported), semen analysis showing azoospermia, pH < 7.0, seminal fructose <13 μmol/ejaculation and GPC <2 μmol/ejaculation (de Souza et al., 2018; Bieth et al., 2021). Other tissue abnormalities in the genitourinary system, such as seminal vesicles and kidneys, can be used as supportive evidence for the diagnosis of CBAVD, but they are not essential diagnostic evidence for the diagnosis of CBAVD. The differential diagnosis of CBAVD involves various male infertility diseases that can cause azoospermia, including obstructive azoospermia and nonobstructive azoospermia. The main difference is that except for CBAVD, all azoospermia-related infertility diseases have evidence of the existence of the vas deferens.

## Treatment and ART Outcomes

From the perspective of andrology, the primary treatment purpose of CBAVD is to solve the problem with the goal of enabling the conception of genetic offspring, and CBAVD is often discovered and diagnosed because of long-term male infertility. With the development of sperm extraction technology and ART, CBAVD patients can achieve fertility with the aforementioned two technologies (Elhanbly et al., 2015; Fang et al., 2020; Jixiang et al., 2021). For CBAVD patients, the first step of ART methods, such as intracytoplasmic sperm injection (ICSI), is the procedure for obtaining sperm. At present, sperm extraction techniques include mainly microsurgical epididymal sperm aspiration (MESA), testicular sperm extraction (TESE), and percutaneous epididymal sperm aspiration (PSEA) (Meniru et al., 1997; van Wely et al., 2015). Although the sperm obtained by these techniques combined with ICSI can enable CBAVD to obtain offspring, there are different reports on the impact of these techniques on the ICSI outcome. Regardless of whether the sperm originates from the testis or the epididymis, it the fertility rate, clinical pregnancy rate and miscarriage rate are the same in obstructive azoospermia (Kamal et al., 2010). However, it has been reported that in patients with CBAVD, extraction of sperm from the epididymis via MESA has higher live birth, clinical and ongoing pregnancy rates than sperm extracted from the testis *via* TSEA (Llabador et al., 2015; van Wely et al., 2015). The reason for this difference may be related to the following factors.

### Spermatogenesis State

One study found that among CBAVD patients undergoing TESE, some have normal spermatogenesis, while others have hypospermatogenesis (Llabador et al., 2015). Compared with normal spermatogenesis, hypospermatogenesis has a lower pregnancy rate (65.6 vs. 72.9%) and embryo cleavage rate (88.6 vs. 92.1%) (Llabador et al., 2015). Although the number of sperm required for ICSI is low, patients with hypospermatogenesis can provide enough sperm for ICSI. Therefore, lack of attention to the spermatogenesis status of patients in CBAVD studies of ART outcomes may be an important contributing factor to inconsistencies in the results of ART outcome analyses.

Age is related to the state of spermatogenesis. It has been shown that the sperm status obtained by PSEA in CBAVD patients is affected by age. Among patients aged <30 years, 30–40 years and >40 years, the sperm number, variability, and normal sperm rate gradually decreases with age, and the pregnancy rate and take-home baby rate also decrease. Accumulated evidence shows that semen parameters in normal males gradually become abnormal with age, including a decrease in semen concentration and semen quantity and an increase in the deformity rate and DNA fragment rate (Vaughan et al., 2020; Gao et al., 2021; Ramirez et al., 2021; Zhang et al., 2021). In these studies, age grouping was narrow, suggesting that small increases in age lead to semen parameter abnormality. In CBAVD patients, significant changes in semen parameters are evident with an age gap of only 10 years and the small age gap in CBAVD patients has a significant impact on ART outcome. Therefore, age differences among CBAVD populations in different studies, which may not be significant between groups, may lead to different ART outcomes due to variation in spermatogenesis.

### Genetic Mutation Status

CFTR is expressed in almost all germ cells and is involved in the process of spermatogenesis (Teixeira et al., 2013), and its mutation may cause abnormal spermatogenesis and lead to attenuated sperm function, such as sperm fertilizing capacity (Xu et al., 2007). CMA3 reflects the chromatin concentration. The higher the CMA3 level in sperm is, the lower the fertilization rate will be. One study showed that compared to those in the sperm of non-CBAVD obstructive azoospermia patients, CMA3 levels were highest in the sperm of CBAVD patients (Ramos et al., 2004), suggesting that sperm function was impaired in CBAVD. Additionally, compared with patients with non-CBAVD obstructive azoospermia, the frequency of CFTR mutations in CBAVD patients was higher, and the rate of miscarriage and stillbirth was higher (Lu et al., 2014). Consistently, another study confirmed that compared with CBAVD, the acquired cause of obstructive azoospermia, such as non-CBAVD obstructive azoospermia, has a higher pregnancy rate and lower abortion rate (Nicopoullos et al., 2004). In addition, compared with non-*CFTR* mutations, *CFTR* mutations can lead to higher abortion and stillbirth rates (Lu et al., 2014). However, a study on ICSI outcomes in CBAVD patients without *CFTR* gene mutations found that the risk of chromosomal abnormalities after ICSI in CBAVD patients was similar to that in normal men, and the pregnancy success rate was also similar, even under the premise of limited spermatogenesis (Viville et al., 2000). Therefore, *CFTR* mutations in CBAVD patients may cause dysfunction of sperm and/or spermatids and finally disrupt ART outcomes. Although there is a lack of research on the influence of other genetic mutations in CBAVD on the ART outcome, genetic mutations may affect the ART outcome by impairing sperm/spermatid function. Therefore, inconsistencies in the genetic mutation status may lead to different ART outcomes reported in different CBAVD populations.

### Genetic Mutation Types

The types of mutations in CBAVD patients also seem to be related to ART outcomes. In a study of a European health population, among three *CFTR* mutants related to CBAVD, Met470Val in exon 10 and the (TG)m and polyT repeat polymorphisms in intron 8, were counted, and only the Met470 allele was related to a lower birth rate (Kosova et al., 2010). The different spectrum of genetic mutants and the different types of genetic mutations in different CBAVD populations explain the inconsistent ART outcomes in some studies about CBAVD.

In short, the ART outcomes of CBAVD patients may not be related to sperm retrieval technology but has important relationships with other factors, including age gap, spermatogenesis state, genetic mutation status and genetic mutation types.

## Genetic Counseling

*CFTR* mutation is the main cause of CBAVD. Several CBAVD family studies have found that when the fathers carries a *CFTR* mutation, his offspring can inherit the mutation and suffer from CF(Patrizio et al., 1993; Wong et al., 2004; Van Hoorenbeeck et al., 2007; Yang et al., 2018; Gaikwad et al., 2020), indicating that *CFTR* mutants in CBAVD patients have a certain risk of being transmitted to offspring. It is worth mentioning that although CFTR gene mutation is the main pathogenic factor underlying CBAVD occurrence, not all CBAVD patients have *CFTR* gene mutations (Patat et al., 2016; Yang et al., 2017). In CBAVD patients with CFTR mutations, not all mutations can be passed on to offspring, and not all mutations passed on to offspring can cause CBAVD or CF. Only pathogenic CFTR mutations are transmitted to offspring can cause the diseases. Therefore, for CBAVD patients, it is necessary to conduct genetic counseling and detection to determine whether there is a pathogenic *CFTR* mutation and the risk degree of passing it to offspring. Some researchers have suggested that all CBAVD patients should be tested for *CFTR* mutations (Cuppens and Cassiman, 2004). However, considering the economic burden to each patient and the limitations of regional medical resources, it is impossible to perform *CFTR* mutation testing for all CBAVD patients. Therefore, when a CBAVD patient has the following clinical characteristics, we should highly suspect that this patient may have a *CFTR* gene mutation, and *CFTR* mutation screening for the following should be carried out before ART: 1. manifestations related to CF (Samli et al., 2006); 2. family history of CF (Cruger et al., 2003); and 3. concomitant renal hypoplasia, semen volume ≤1.0 ml and pH < 7.0 (Daudin et al., 2000).

Among studies on *CFTR* mutations in CBAVD, many pathogenic *CFTR* mutants, such as p. Arg117His, I556V, IVS9-5T allele and F508del, have been identified (Cuppens et al., 1998; Uzun et al., 2005; Lu et al., 2013; Thauvin-Robinet et al., 2013). However, due to differences in medical resources and detection technology in various regions, *CFTR* mutants with high frequency in the CBAVD patient’s region should be screened first when the CBAVD patient is highly suspected of having a CFTR mutation. If the first mutation detection result is negative, the detection range of *CFTR* mutants should be expanded. Additionally, we always detect only single *CFTR* mutations in traditional detection methods (Giuliani et al., 2010). However, screening for a mutation in both *CFTR* alleles is helpful to calculate the risk of offspring with CF (Giuliani et al., 2010), especially when the female partner is negative for the *CFTR* mutation. Therefore, we recommend that both *CFTR* alleles be screened for the mutation.

Previously, it was believed that *CFTR* mutants need to be screened only in CBAVD patients because CBAVD patients with severe *CFTR* mutations have a 50% probability of passing the *CFTR* mutations on to their offspring (Cuppens and Cassiman, 2004), suggesting that it is of great value to detect *CFTR* mutations in CBAVD patients. If the *CFTR* mutation screening result of the CBAVD patient is negative, there is no need to screen the female partner for the *CFTR* mutation. However, it has been found that *CFTR* mutations in the female partner may not necessarily be accompanied by significant clinical manifestations (Cuppens and Cassiman, 2004), making her status easy to ignore. However, *CFTR* mutants in female partners can significantly disrupt ART outcomes and increase the transmission risk of *CFTR* mutations to offspring (Lewis-Jones et al., 2000). One study showed that the presence of *CFTR* mutations in female partners increased the risk of miscarriage (Peleg et al., 2011). At the same time, under the premise that both the CBAVD patient and his spouse have *CFTR* mutations, the risk of CF in the offspring (1 in 25) is 25 times that of the normal population (1 in 2,500) (Cuppens and Cassiman, 2004). However, if the female is CF-negative or has no *CFTR* mutations, the probability of children getting CF or CBAVD will be reduced to 1 in 960 (Lewis-Jones et al., 2000). Therefore, *CFTR* mutant screening in female partners of CBAVD patients cannot be ignored. Ideally, if conditions are sufficient, regardless of whether CF occurs, it is recommended that the CBAVD patient and his spouse undergo *CFTR* mutation screening to thoroughly assess the risk of offspring with CF.

With in-depth research on the genetic etiology of CBAVD patients, mutations in other genes, such as *ADGRG2,* may be considered in addition to the *CFTR* gene because not all regions are dominated by *CFTR* mutations in terms of their CBAVD populations. Among CBAVD sequencing results reported in different countries, *ADGRG2* mutation in cases without *CFTR* mutation may occur in addition to *CFTR* mutation (Patat et al., 2016; Khan et al., 2018; Yuan et al., 2019; Pagin et al., 2020). In short, for CBAVD patients and spouses, genetic counseling is necessary to ensure the birth of healthy offspring.

## Conclusion

CBAVD is a male infertility disease. Mutations in genes such as *CFTR* and *AGDRD2* are the main pathogenic factors of CBAVD. Semen extraction technology and ART can enable CBAVD patients to achieve fertility. Genetic mutations in CBAVD patients can affect spermatogenic function and sperm quality, thereby interfering with the ART outcome. In addition, there is a risk of passing on pathogenic genetic mutations to offspring. Therefore, genetic counseling for CBAVD patients and their spouses is necessary to produce healthy offspring.
